# Data connectivity: A critical tool for external quality assessment

**DOI:** 10.4102/ajlm.v5i2.535

**Published:** 2016-10-17

**Authors:** Ben Cheng, Brad Cunningham, Debrah I. Boeras, Patron Mafaune, Raiva Simbi, Rosanna W. Peeling

**Affiliations:** 1 International Diagnostics Centre, London School of Hygiene & Tropical Medicine, London, United Kingdom; 2 SystemOne, Johannesburg, South Africa; 3 Ministry of Health and Child Care, Harare, Zimbabwe

## Abstract

Point-of-care (POC) tests have been useful in increasing access to testing and treatment monitoring for HIV. Decentralising testing from laboratories to hundreds of sites around a country presents tremendous challenges in training and quality assurance. In order to address these concerns, companies are now either embedding connectivity in their new POC diagnostic instruments or providing some form of channel for electronic result exchange. These will allow automated key performance and operational metrics from devices in the field to a central database. Setting up connectivity between these POC devices and a central database at the Ministries of Health will allow automated data transmission, creating an opportunity for real-time information on diagnostic instrument performance as well as the competency of the operator through external quality assessment. A pilot programme in Zimbabwe shows that connectivity has significantly improve the turn-around time of external quality assessment result submissions and allow corrective actions to be provided in a timely manner. Furthermore, by linking the data to existing supply chain management software, stock-outs can be minimised. As countries are looking forward to achieving the 90-90-90 targets for HIV, such innovative technologies can automate disease surveillance, improve the quality of testing and strengthen the efficiency of health systems.

## Introduction

Diagnostic testing has traditionally been conducted in a laboratory setting, and requires highly-skilled operators working in strictly-controlled laboratory environments. The recent availability of point-of-care (POC) technologies allows for a shift from a centralised to a decentralised model of testing. Such decentralisation of testing can offer a significant increase in access to much needed diagnostics for populations in peri-urban, rural and other hard to reach areas, which will be needed to achieve the UNAIDS 90-90-90 target.^1^ Furthermore, the scale up of quality POC testing can help strengthen health systems, especially in rural areas.^2^

The wide deployment of POC tests at hundreds or even thousands of sites raises a big challenge for Ministries of Health to track instruments and monitor the quality of the test and the testing. In order to address these concerns, numerous connectivity (connected diagnostic) initiatives and interventions have been undertaken in an attempt to ensure that the implementing institution is able to keep track of, at minimum, key performance and operational metrics for devices in the field. These initiatives have had mixed success in implementation due to a variety of reasons. Companies are now either embedding connectivity in their new diagnostic instruments or providing some form of channel for electronic result exchange.

Zimbabwe, for example, has placed 346 Alere Pima™ POC CD4 devices in hard to reach areas throughout the country. At present, 114 of these instruments are connected via Alere 3G modems to the Clinton Health Access Initiative’s in-country POCLabs platform in conjunction with the National Microbiology Reference Laboratory. The Ministry of Health has been receiving scant data from the devices, as is shown in the snapshot provided in Table 1. This is likely due to a combination of reasons including: lack of awareness regarding sending of data, insufficient 2G/3G signal, SIM cards with insufficient/expired airtime, and other SIM issues.

**TABLE 1:** Snapshot of reporting frequency from connected devices in Zimbabwe, 2016.

Last data received	Number of sites reporting
0–10 days	4
11–30 days	21
31–60 days	9
61–90 days	8
90 days or more	72
**Total number of instruments**	**114**

## External quality assessment challenges for point-of-care testing

External quality assessment (EQA) is a critical component of a laboratory quality assurance and management system.^3^ EQA for POC testing has the potential to provide a snapshot of quality, both in terms of the diagnostic instrument performance as well as the competency of the operator. The implementation of POC testing in rural and peri-urban settings, however, presents tremendous challenges in the organisation of an EQA programme. These challenges include:

- The scale of providing EQA samples to hundreds of sites within a country, requiring the preparation or procurement of a large number of proficiency testing panels and the means to distribute them.
- POC tests are typically operated by non-laboratory staff who are not familiar with a laboratory ‘culture’ of quality or EQA.
- Poor response rates and delay in reporting weaken the value of EQA programmes.

### How can connectivity improve the quality of testing?

Connectivity for POC tests and other medical devices can be defined as an initiative that can improve patient flow, increase the quality of testing, and improve patient outcomes. Connectivity should be viewed as a complementary approach to EQA. It can provide continuous quality monitoring, as all of the data generated by the instrument can be analysed as an average of a series of aggregated, non-unique events, whereas EQA is a series of controlled events, with known inputs and known outputs. Connectivity can be used not only to monitor patient data such as diagnostic results and response to treatment, but it can also be used to capture operational data from the device and provide key performance and quality indicators.

**FIGURE 1:**
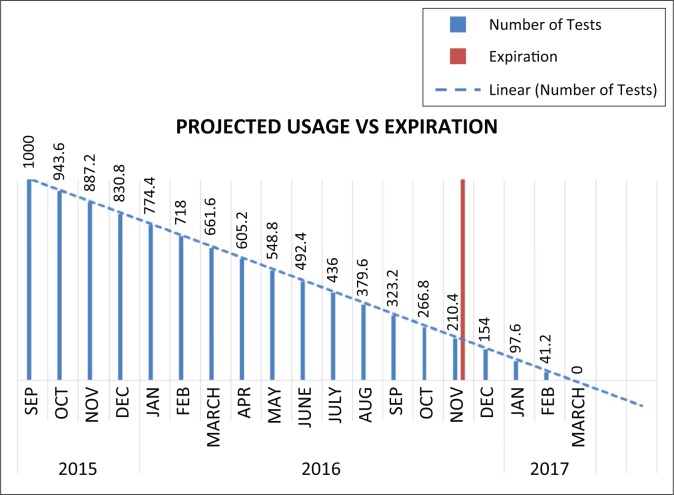
Using connectivity to monitor utilisation and predict stock-outs.

Most POC instruments now include methods to exchange data electronically. This can be via a port (e.g., USB, Ethernet, serial), or via embedded GSM/3G capabilities within the POC device. The diagnostics industry is beginning to take full advantage of these capabilities with a number of ecosystems being constructed around the consumption of this data. Various stakeholders have distinct, and well-defined needs for the different data sets and subsets. Broadly, the data can be categorised into clinical data, which includes patient-identifiable information, and operational data. Operational data is defined as a subset of the clinical data which is de-identified. These systems allow Ministries of Health to receive information and derive intelligence, in real-time, from any testing facility throughout a country into one central database. This allows Ministries of Health to have daily monitoring of the performance of the devices, the test results and the competency of the operators from each site. Furthermore, connectivity can help improve forecasting and prevent stock-outs based on the utilisation rate at each facility, as shown in Figure 1.

Operational data can be used by instrument manufacturers, Ministries of Health, funders, trainers, and supply chain managers. Diagnostic data can be used for patient management by nurses, clinicians and epidemiologists and stored in a laboratory information system and/or a central data warehouse.^4^ These informatics systems are continually expanding and innovating features as the ecosystems become better defined. One example is the automated detection and transmission of EQA samples from diagnostic devices which is made available, in near-real time, to the EQA providers for analysis.

### Existing point-of-care external quality assessment structure for the Pima CD4 in Zimbabwe

Historically, the Pima CD4 EQA programme in Zimbabwe requires a significant amount of hands-on time for both sample shipping, testing and reporting. The process, briefly, is as follows.

**FIGURE 2:**
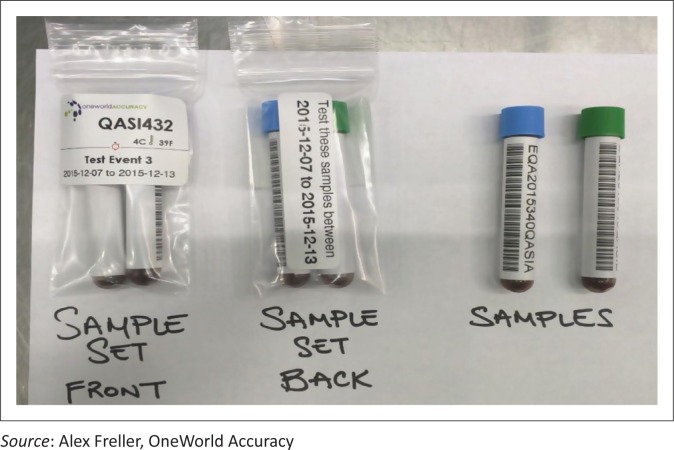
Sample EQA set sent to sites in Zimbabwe.

EQA samples are shipped to a single, central, location for separation and distribution. This facility unpacks the samples and re-packages them for distribution by courier for delivery to the facility (Figure 2). After the samples have been received by the testing facility, they are required to be completed before the scheduled deadline, and the results are reported, via different mechanisms, back to the central facility.

Once the EQA deadline closes, the central facility captures all of the received results, first on paper for central collection, and then via a web-form on the EQA provider’s informatics platform, for all participating sites. Once the EQA results have been captured and submitted, the EQA provider is able to analyse the results and report these, with any corrective action suggestions, back to the central facility. At this point, the central facility is, again, responsible for separating the individual performance reports for the facilities and re-distributing these, along with any corrective actions, back to the participants. This whole process usually takes at least 12 weeks.

For connected devices, it is clear that a much simpler and more efficient workflow can be leveraged to optimise the EQA process. POC tests connected to an informatics system capable of distinguishing EQA from routine samples via the sample ID can dramatically help improve turnaround time for EQA results, thereby allowing for action to be taken if necessary. Furthermore, there will be significantly fewer transcription errors and data analysis will be simplified.

## Proof of concept for an automated external quality assessment programme in Zimbabwe

**FIGURE 3:**
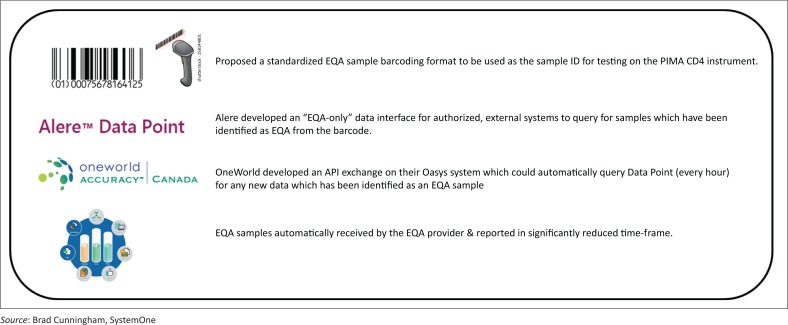
Steps involved in the automated EQA reporting proof-of-concept, Manicaland Province, Zimbabwe, November 2015.

An automated EQA proof-of-concept pilot for CD4 counts was conducted in November 2015 in Manicaland Province in Zimbabwe. Several issues had to be resolved among different partners before the proof-of-concept testing could be initiated. A standardised barcoding format had to be agreed upon for use as the sample ID for testing on the Pima CD4 instrument. The barcode standardisation was necessary so that the informatics systems would be able to differentiate EQA samples from patient results to ensure that no patient data is transmitted beyond the clinical boundaries defined in these structures (Figure 3).

Alere, the developer of the Pima POC CD4 device developed an ‘EQA only’ data-store and interface for its centralised data warehouse, Data Point. This allows for authorised, external systems to query Data Point for samples that have been identified as EQA samples from the barcode. For this pilot, the EQA provider, Oneworld Accuracy, developed an application program interface for their informatics system, OASYS (Oneworld Accuracy System), which would automatically query Data Point every hour for any new data that has been identified as an EQA sample. All new data identified as EQA are then immediately received by OASYS for storage and processing (both of these application program interfaces have now been included in the production version of both Data Point and OASYS).

The proof-of-concept study only involved one site, the Marange rural health centre. Three sets of EQA samples were delivered to Marange and run by the operator over a three-week period. Oneworld Accuracy received the results from the EQA samples within 60 minutes of these tests being performed on the Pima device, demonstrating that the automated system performed as expected. The next phase was to increase the number of sites to approximately 50 and EQA samples were distributed as part of the routinely-scheduled EQA assessment planned for the end of March 2016.

Due to the perceived impact of this intervention, a similar process is currently being established to automate the reporting of EQA samples (identified by a barcode) from the Cepheid GeneXpert^®^ platform to the relevant EQA provider informatics systems. The proof-of-concept testing for this took place in April 2016 at four sites in Manicaland.

### Post-proof of concept

The proof-of-concept pilot highlighted the potential for improvement in turn-around time from testing to reporting of EQA results, as well as reducing the overall administrative burden of these events. It is recommended that some standard barcoding identification be implemented for all EQA samples for both laboratory and POC instruments. The implementation of a standardised barcode structure for the identification of EQA samples would allow any informatics system (operational or clinical), connected to any laboratory or POC instrument, to identify a sample as an EQA sample and transmit these results, in near-real time, to the relevant EQA provider for analysis and reporting.

In the ideal case, the following workflow could become standard for EQA testing and reporting:

- The EQA provider sends out the sample to the test site.
- The test site receives the sample and tests the sample.
- The results are automatically uploaded to a server upon completion.
- The server identifies the result as an EQA result and forwards the result to the correct EQA provider.
- The EQA provider scores the result and generates the user report.

This proposed workflow reduces the need for the paper transcription of results to be collected centrally, as well as the need for the collected results to be re-transcribed in an electronic format on the EQA provider’s informatics platform, thus reducing errors in both interpretation and transcription.

### Way forward

The automation of the collection and analysis of EQA results from laboratories, especially for POC instruments, can help countries:

- Significantly improve the turn-around time of EQA result submissions and the impact of any prescribed corrective actions in a timely manner.
- Reduce the transcription error rate and improve the quality of the resulting process.
- Eliminate the time-consuming processes involved with EQA result collection, capture and re-distribution.
- Reduce the cost and improve uptake of EQA programmes for both laboratories and POC facilities.

Additional expected outputs from this pilot are:

- Feasibility and acceptability from EQA providers to adhere to a standardised barcode format.
- Willingness for Ministries of Health to have this data (non-patient data) automatically shared between connected systems (and ecosystems) from the instrument to the EQA provider.
- Definition of the role the instrument manufacturer can play in helping countries successfully implement and monitor EQA programmes via manufacturer platforms.

### Conclusion

Connectivity has shown that it is possible for Ministries of Health to have up-to-the-hour information on testing and test results across the country. These results can also be accompanied by EQA results from laboratories, as well as each POC testing site across the country, so that corrective action and refresher training can be provided in a timely manner. These systems can also be twinned to supply chain management software to monitor supplies at each site, providing an automated system for alerts to avoid stock-outs. As countries are looking forward to achieving the 90-90-90 targets for HIV, such innovative technologies can automate surveillance, improve quality of testing and strengthen health systems.
